# A Neural Network Approach for Understanding Patient Experiences of Chronic Obstructive Pulmonary Disease (COPD): Retrospective, Cross-sectional Study of Social Media Content

**DOI:** 10.2196/26272

**Published:** 2021-11-11

**Authors:** Tobe Che Benjamin Freeman, Raul Rodriguez-Esteban, Juergen Gottowik, Xing Yang, Veit Johannes Erpenbeck, Mathias Leddin

**Affiliations:** 1 Roche Pharma Research and Early Development Pharma Research and Early Development Informatics Roche Innovation Center Basel, F. Hoffmann–La Roche Ltd Basel Switzerland; 2 wordup development AG CH-8006 Zurich Switzerland; 3 Roche Pharma Research and Early Development Pharma Research and Early Development Informatics Roche Innovation Center Little Falls, F. Hoffmann–La Roche Ltd Little Falls, NJ United States; 4 Roche Pharma Research and Early Development Immunology, Infectious Diseases and Ophthalmology Discovery and Translational Area Roche innovation Center Basel, F. Hoffmann–La Roche Ltd Basel Switzerland

**Keywords:** outcomes research, natural language processing, neural networks (computer), social media, exercise, sleep deprivation, social media listening, drug development

## Abstract

**Background:**

The abundance of online content contributed by patients is a rich source of insight about the lived experience of disease. Patients share disease experiences with other members of the patient and caregiver community and do so using their own lexicon of words and phrases. This lexicon and the topics that are communicated using words and phrases belonging to the lexicon help us better understand disease burden. Insights from social media may ultimately guide clinical development in ways that ensure that future treatments are fit for purpose from the patient’s perspective.

**Objective:**

We sought insights into the patient experience of chronic obstructive pulmonary disease (COPD) by analyzing a substantial corpus of social media content. The corpus was sufficiently large to make manual review and manual coding all but impossible to perform in a consistent and systematic fashion. Advanced analytics were applied to the corpus content in the search for associations between symptoms and impacts across the entire text corpus.

**Methods:**

We conducted a retrospective, cross-sectional study of 5663 posts sourced from open blogs and online forum posts published by COPD patients between February 2016 and August 2019. We applied a novel neural network approach to identify a lexicon of community words and phrases used by patients to describe their symptoms. We used this lexicon to explore the relationship between COPD symptoms and disease-related impacts.

**Results:**

We identified a diverse lexicon of community words and phrases for COPD symptoms, including gasping, wheezy, mucus-y, and muck. These symptoms were mentioned in association with specific words and phrases for disease impact such as frightening, breathing discomfort, and difficulty exercising. Furthermore, we found an association between mucus hypersecretion and moderate disease severity, which distinguished mucus from the other main COPD symptoms, namely breathlessness and cough.

**Conclusions:**

We demonstrated the potential of neural networks and advanced analytics to gain patient-focused insights about how each distinct COPD symptom contributes to the burden of chronic and acute respiratory illness. Using a neural network approach, we identified words and phrases for COPD symptoms that were specific to the patient community. Identifying patterns in the association between symptoms and impacts deepened our understanding of the patient experience of COPD. This approach can be readily applied to other disease areas.

## Introduction

Online content made public by patients in blogs and on forum platforms provides detailed first person accounts of the lived experience of disease [1,2]. These communications from patients use a diverse vocabulary of words and phrases for disease symptoms [3]. Online content is conveyed in the patient’s own voice and is contributed in the ecological context of day-to-day life [4], namely in the sharing of experiences with other members of the patient and caregiver community. Analysis of these online communications enables a patient-centric approach to understanding disease impact.

A systematic understanding of the language used by patients to describe their symptoms has important clinical implications, not least being the need to acquire accurate patient anamneses and respond to care needs [5]. Dreisbach et al [6] note that the use of normalized medical vocabularies supports a systematic approach to identify terms for clinical and subclinical symptoms. This approach enables the identification of community terms that, while not belonging to a traditional medical lexicon, denote respiratory dysfunction unambiguously.

Many researchers use interviews, focus groups, and patient advisory boards with a goal of observing patient experiences. These approaches enable direct observation of the patient; however, they tend to be a burden to patients [7]. Moreover, interviews and focus groups are generally limited to cohorts of just a few patients, and the results are qualitative in nature.

In contrast, machine learning and related computational techniques offer a means to analyze online content at scale. Current state-of-the-art approaches using neural network architectures are being deployed to map patient community terms onto controlled medical [8] and pharmaceutical vocabularies [3]. However, these approaches are anchored in a defined lexicon of scientific terms, thus compromising patient centricity. In a patient-centric approach, our understanding of disease should instead be anchored to patients’ self-reported topics [7], as observed in the ecological context of daily life [4], and not exclusively anchored to expert medical thinking, as expressed in a scientific lexicon.

We address this limitation with a novel approach based on a neural network, specifically a word embedding [9], to identify words and phrases that patients with chronic obstructive pulmonary disease (COPD) use to describe their experiences of living with the disease. Unlike traditional neural network approaches, a word embedding is not trained on any specific set of scientific keywords [10,11].

We use the word embedding to identify a diverse lexicon of hundreds of COPD-related words and phrases from the context in which words appear in a text. Next, we use that lexicon to extract all mentions of words and phrases relating to COPD symptoms and disease impacts from a large corpus of social media text. Once extracted, we can analyze the relationship between COPD symptoms and disease impacts at scale.

The quantitative analysis of this diverse community lexicon reveals insights [6] about the lived experience of COPD. These insights can contribute positively to the development of effective medical treatments that are, from the patient’s perspective, fit for purpose [12].

## Methods

### Ethics

This work is compliant with ethical guidelines for the collection and analysis of user-generated content on open internet platforms. Data were downloaded only from open health social networking sites and communities. No information from restricted data areas has been downloaded (ie, content that requires an ID or password for access). No aggregation or enrichment of data on an individual has been performed. Extracts used for exemplary purposes were carefully paraphrased to protect the privacy of individuals.

### Data Availability

All social media content included in our analysis was sourced from open social networking sites and communities. Terms and conditions apply to the availability of the original social media data. The sources used in this study can be made available upon request. Example texts shown in this manuscript have been rephrased to prevent de-anonymization of the individuals included in our analysis.

### Neural Network Methodology

We trained the neural network on a corpus of 1.1 million words sourced from 22 individual blogs and online forums (Multimedia Appendix 1). We used the skip-gram negative sampling variant of the word2vec neural network algorithm described by Mikolov et al [9] to discover community words and phrases for disease symptoms. Briefly, the neural network model was trained to predict context words that appear in close proximity with symptom keywords in the corpus text.

The resulting word embedding captured semantic and syntactic features of each unique word in the text corpus. Neighboring vocabulary items in the embedding will likely share semantic and syntactic features in common. We then used cosine similarity as a metric to probe the word embedding model for words and phrases that share common meanings. This makes it possible to build and expand a lexicon of community terms for each main COPD symptom type in a systematic and repeatable manner (Table S1 in Multimedia Appendix 1).

We started our search for community words and phrases for COPD symptoms with a small seed lexicon that included breathlessness, cough, and sputum. This seed lexicon was sourced from MeSH terms from the US National Library of Medicine (NLM) [13] and from the NLM health information website for the layperson, MedlinePlus [14]. These 3 seed terms correspond to key pathophysiological manifestations of COPD, namely small airway fibrosis, emphysema, which refers to a destruction of the lungs’ alveoli, and mucus hypersecretion [15-17].

We used the same approach to search for community words and phrases describing the impact of COPD on daily life. The seed terms for disease impacts include anxiety, depression, fatigue, pain, and exercise. We then scanned the entire corpus to detect posts in which COPD symptoms co-occur with mentions of disease-related impacts. Our analysis explored the relationship between specific symptoms and each of the main disease impact topics.

## Results

Using the cosine similarity metric to probe the word embedding model, close neighbors of the symptom seed term breathlessness included gasping, wheezy, and the phrase pursed-lip (Table S1 in Multimedia Appendix 1). The phrase pursed-lip is noteworthy as it refers to a technique, called pursed-lip breathing, used in pulmonary rehabilitation. Specifically, pursed-lip breathing is used to manage anxiety associated with breathlessness [18]. Words and phrases neighboring the seed term sputum include mucus-y, phlegm, clear mucus, and muck, as well as common misspellings of phlegm.

Probing the word embedding model with the seed term exercise, we found walk and the phrases low impact and difficulty exercising (Table S1 in Multimedia Appendix 1). These community terms are, as we might expect, for a relatively aged and exercise-limited patient cohort [19]. Manual inspection of individual excerpts from the corpus featuring symptom keywords further confirmed the relevance of these keywords (Table S4 in Multimedia Appendix 1).

Summing the number of mentions corresponding to each symptom lexicon across the entire corpus (Table S2 in Multimedia Appendix 1), the breathlessness lexicon was mentioned most frequently (mentioned in 10.49% [413/3938] of posts), followed by the lexicon for cough (270/3938, 6.86%) and, finally, mucus hypersecretion (159/3938, 4.04%).

**Figure 1:**
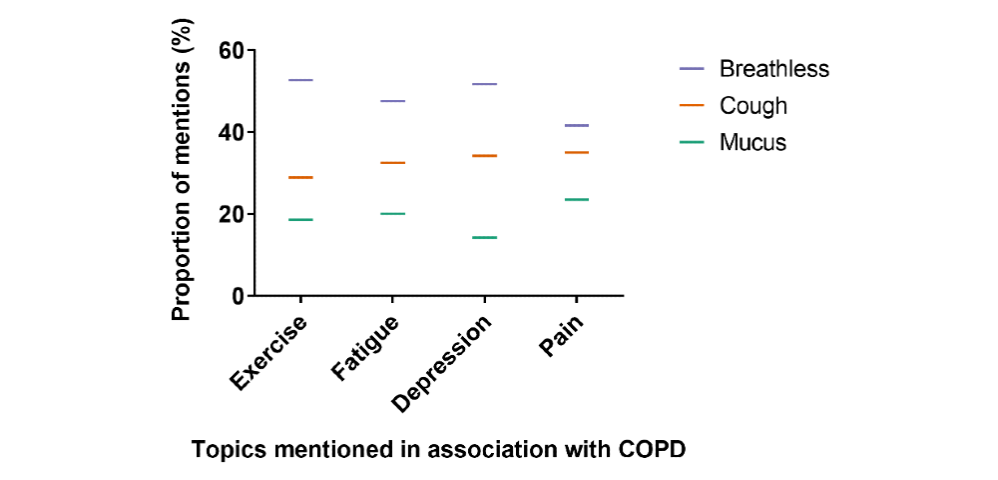
Topics co-occurring with symptom mentions in the same post.

Leveraging these distinct lexicons of symptoms and disease impacts (Table S3 in Multimedia Appendix 1), we were able to explore the relationship between specific symptoms and each of the main disease-impact topics. Figure 1 examines posts in which COPD symptoms co-occurred with mentions of disease-related impacts. The analysis shows that breathlessness was the symptom most frequently mentioned in association with the 4 main topics and impacts considered. The most frequent disease impact associated with COPD symptoms was fatigue, followed closely by self-reports of anxiety and depression.

**Figure 2:**
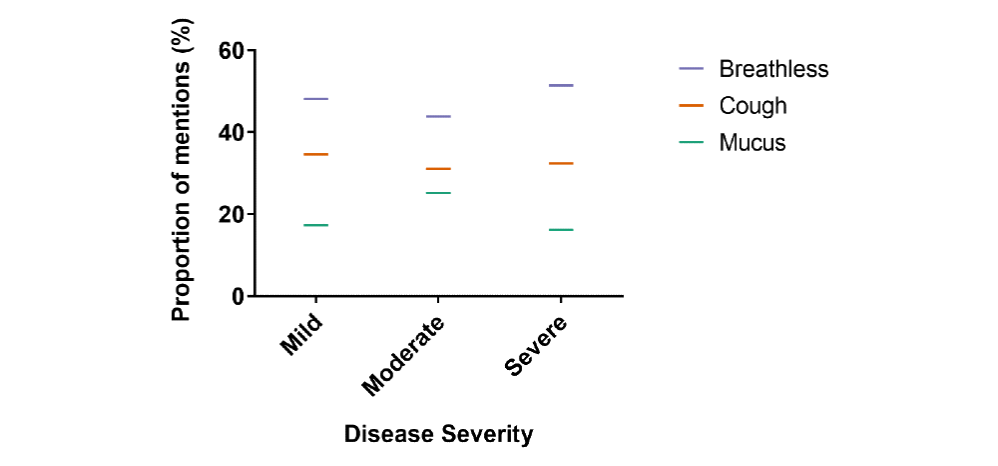
Relationship between the symptoms of chronic obstructive pulmonary disease and disease severity.

Breathlessness and cough followed a broadly similar trend, while the trend in the co-occurrence between mucus and the 3 disease severity levels was distinctive (Figure 2). The co-occurrence between mucus and mild severity was lower than that between mucus and moderate disease severity, inverting the relationships observed for breathless and for cough. Taken together, it was apparent that there was an association between mucus and moderate disease severity that distinguished mucus from the symptoms breathlessness and cough.

By applying principal component analysis (PCA), we visualized semantic relationships [20,21] between each symptom lexicon and a mapping of the psychological salience of these symptoms. PCA arranged data points corresponding to individual words and phrases on a 2D map [20] (see Multimedia Appendix 1 for further details). Our PCA results showed that words and phrases belonging to the 3 symptom lexicons were arranged in 3 distinct clusters on this map (Figure S1 in Multimedia Appendix 1).

By adding a lexicon of affective states such as feel depressed and be embarrassing to the PCA map, we could explore the psychological salience of these symptoms. The lexicon of affective states also appeared as a distinct cluster on the map and was positioned closest to the cough symptom cluster. The mucus cluster was displaced further away from the cluster of affective states than the cough cluster. Note, however, that the cough and mucus clusters were aligned along a single axis with respect to the cluster of affective states.

## Discussion

### Principal Findings

Our findings demonstrate the potential to deploy advanced analytics in the search for disease-related insights from hundreds of patients and many thousands of self-reports published online.

By probing a word embedding model trained on a corpus of online content contributed by COPD patients, we found a lexicon of community terms expressing a broad range of topics and meanings (Table S1 in Multimedia Appendix 1). Many terms found this way were related to COPD in a direct and intuitive fashion. And some terms revealed associations with unexpected, yet highly relevant topics (eg, pursed-lip) [18]. This term relates to the pursed-lip technique for managing anxiety associated with breathlessness.

The finding that breathlessness was the most frequently mentioned symptom accorded with medical consensus. As stated by the internationally recognized guidelines of the Global Initiative for Chronic Obstructive Lung Disease (GOLD) [15,22], a decline in lung capacity, in combination with other disease-specific symptoms [23,24], forms the basis of a clinical diagnosis of COPD, and measurements of lung function and lung volumes are used to monitor disease progression [17].

In agreement with recent social media studies of COPD patients, our results highlight mucus hypersecretion as an important COPD symptom [25,26]. Compared with breathlessness and cough, mucus terms co-occur with mentions of moderate disease and co-occur less often with mild or severe disease. Similarly, when compared with breathlessness and cough, mucus symptoms were mentioned relatively less frequently when patients reported affective impacts of COPD such as depression.

These distinct associations relating to mucus hypersecretion were corroborated by a novel analysis using PCA to map the psychological salience of the 3 COPD symptoms. Relative to breathlessness and cough, mucus symptoms were mapped furthest from the affective impacts of COPD, suggesting that mucus has the weakest association with perceived affective impacts of the disease.

Mucus symptoms were mentioned at less than half the frequency that breathlessness was mentioned in the corpus. This finding is consistent with the GOLD report and reports indicating that not all COPD patients experience mucus hypersecretion as a symptom of their disease and that mucin concentrations are lower in COPD versus other obstructive lung diseases like cystic fibrosis or bronchiectasis [27]. And yet mucus hypersecretion is an important clinical factor in COPD. For example, mucus symptoms can motivate patients to take timely action against life-threatening respiratory infections [28]. Hypersecretion also drives cough symptoms and expectoration [15].

Without these advanced analytics, our insights about mucus symptoms would have been obscured by the overall dominance of breathlessness and cough symptoms mentioned in the corpus. Examining the co-occurrences between symptoms and disease impacts informed a deeper understanding of disease burden. The approach was able to quickly and accurately identify patient populations whose experience was especially impacted by a particular symptom, adding greater potential for personalization.

This approach can ultimately guide clinical development in ways that ensure that future treatments are fit for purpose from the patient’s perspective [12] and from the perspective of patients’ perceived treatment needs.

### Limitations

The forum content we included in the corpus had been posted anonymously and so we were unable to verify any bias arising from the demographics of forum contributors. Beyond the general guidance posted online by forum moderators, we could not explore biases introduced by a moderator removing posts from the forum.

We can expect a degree of clinical inaccuracy in the contributions posted by individuals who may not have formal medical training. Furthermore, the anonymity of social media makes it all but impossible to determine whether a post is authored by a genuine patient or caregiver or by someone merely posing as one. Taken together, any clinical interpretations we make from social media must take these uncertainties into account. However, because every post was manually reviewed, obviously fraudulent content from bots, scammers, and marketers was eliminated.

Despite limitations, the societal benefits that may be gained from large scale analysis of social media content are substantial, as researchers Gleibs et al [29] and Golder et al [30] have noted. The research community should ideally work closely with patients and health care advocates to ensure that people can continue to contribute to online forums and other social media platforms in a way that protects their privacy and ensures they are safe from potentially harmful misinformation.

### Conclusions

Using a novel neural network approach, we demonstrate how online content can be a rich source of insights about the lived experience of COPD. Our findings demonstrate the potential of neural networks to gain a quantitative, patient-focused understanding about how each distinct COPD symptom contributes to the burden of chronic and acute respiratory illness. This approach can be readily applied to other disease areas in which there exists sufficient online content contributed by patients and caregivers.

## Appendix

Multimedia Appendix 1 Further advanced analyses.

## Abbreviations

COPD: chronic obstructive pulmonary disease
GOLD: Global Initiative for Chronic Obstructive Lung Disease
NLM: National Library of Medicine
PCA: principal component analysis
