# Supplementary material for: A Neural Network Approach for Understanding Patient Experiences of Chronic Obstructive Pulmonary Disease (COPD): Retrospective, Cross-sectional Study of Social Media Content
Source: JMIR Med Inform. 2021 Nov 11;9(11):e26272. doi: 10.2196/26272 (PMC8663584; doi:10.2196/26272)
Supplement: Multimedia Appendix 1 [file medinform_v9i11e26272_app1.docx]

## Multimedia Appendix 1. Further advanced analyses.

We conducted a retrospective, cross-sectional study of content manifestly made public online. We sampled 5663 posts, with a vocabulary of 17,811 unique vocabulary items, published between February 2016 and August 2019 from twenty two separate blogs and online forums.

Posts in which COPD symptoms co-occur with mentions of disease-related impacts were read and reviewed manually to confirm that the relevant community terms were present in the appropriate context. Table S1 presents examples of the terms and phrases that COPD patients use to describe their symptoms, as well as the impact that COPD has on their lives.

**Table S1.** Terms and phrases found using neural network. Left column lists a selection of topics relating to the emotional and physical impact of COPD. Terms in bold indicate the seed terms used to build and expand a lexicon of community terms for each topic. Examples shown in the right column are representative of a larger lexicon of community terms identified by probing the word embedding model.

| Topic | Selected terms & phrases found using neural network |
| --- | --- |
| Breathless symptom | Breathless, gasping, wheezy, pursed lip |
| Cough symptom | Cough, coughed up, huff cough, persistent cough |
| Sputum symptom | Mucus, mucus-y, phlegm, sputum, clear mucus, muck |
| Exercise topic | Exercise, walk, brisk, stride, low impact, difficulty exercising |
| Fatigue topic | Fatigue, tiredness, nap, weak, fatigue monster |
| Depression and anxiety | Anxiety, frightening, upset, depression |
| Pain and discomfort | Pain, sore, breathing discomfort, very uncomfortable, ache |
| Mild disease | Discomfort, achy, breathing discomfort, lung discomfort |
| Moderate disease | Flare-up, flareups, flares |
| Severe disease | Hospitalized, coma, unresponsive, paramedics, intubated |

Table S2 shows the number of times that each of the three types of COPD symptoms are mentioned in the corpus. Table S3 shows the number of times that each of the three types of COPD impacts are mentioned in the corpus. Exercise is the most frequently mentioned impact topic, followed by the impact topic anxiety and depression. The next most frequently mentioned topic is fatigue and finally, pain.

**Table S2.** Number of times patients mentioned community items from each COPD symptom and disease-impact lexicon aggregated across the entire corpus. Each lexicon contains the seed term from NLM’s MeSH and MedlinePlus, close neighbours of these terms as defined using cosine similarity, and finally misspellings of these terms. Note that if a post mentions multiple symptom types, for example coughing and mucus, this post is included in the calculation for both cough symptoms and mucus symptoms. However, this same post is counted once only in the aggregate of all posts (n=3938).

| Topic | Aggregated mentions across corpus, n (%) |
| --- | --- |
| Breathless lexicon | 413 (10.5) |
| Cough lexicon | 260 (7.6) |
| Mucus lexicon | 159 (4.0) |
| Aggregate of all symptom posts | 552 (14.0) |

Note the terms and phrases corresponding to disease severity. Mild disease severity terms include *discomfort* and *achy*. Severe disease terms include *unresponsive* and *intubated*, namely descriptions of cardiopulmonary resuscitation and respiratory intubation. An intermediate level of disease severity is defined between discomfort and severe events. Vocabulary items corresponding to this intermediate level include mentions of flare-ups and exacerbations (Table S1).

Manual review of posts mentioning exacerbations and a specific symptom confirmed that most posts describe events that are more severe than mild discomfort, yet did not involve strongly severe events such as emergency care procedures and unscheduled admissions to hospital.

We calculate the aggregate number of symptom mentions across the entire corpus of social media text. Each symptom lexicon contains the seed term from NLM’s MeSH and MedlinePlus vocabularies, close neighbours of these terms as defined using the cosine similarity, and finally the misspellings of these terms that we find by probing the word embedding model for variant spellings (eg, ‘phlem’ as a misspelling of phlegm).

Note that the total number of symptom mentions in Table S1 is not the sum of the three individual symptom types. If a post mentions multiple symptom types, for example coughing and mucus, this post will be included in the calculation for both cough symptoms and mucus symptoms. Furthermore, if a post contains multiple mentions of the same symptom, the number of mentions recorded for that post is defined as one.

**Figure S1**. Embeddings projected onto two dimensions using principal component analysis (PCA). Each 300 dimensional embedding vector is represented as a single point. Phrases relating to the primary symptoms of COPD, namely dyspnea (breathlessness), are colour coded purple. Phrases relating to cough are coloured red and sputum phrases are green. Phrases in black correspond to affective states. Some text labels are omitted for the purpose of legibility.


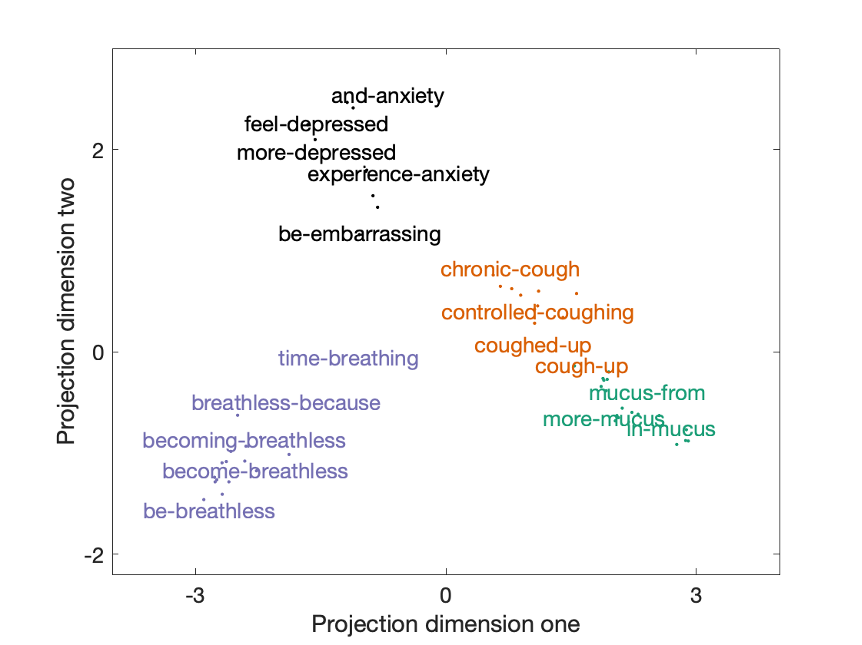


**Table S3**. Number of times patients mentioned community items from each COPD disease-impact lexicon aggregated across the entire corpus (n=3938). Methods of calculation same as Table S2.

| Topic | Aggregated mentions across corpus, n (%) |
| --- | --- |
| Exercise | 613 (15.6) |
| Depression and anxiety | 435 (11.0) |
| Fatigue | 336 (8.5) |
| Pain | 286 (7.3) |
| Aggregate of all disease impact posts | 1093 (27.8) |

### Visualizing Relationships Between Vocabulary Items in the Embedding Model Using Principal Component Analysis

We use principal component analysis (PCA) to visualise the embeddings of words and whole-phrase vocabulary items. We leverage PCA to project the original 300 dimensional embedding vectors, corresponding to individual vocabulary items, onto a two-dimensional map. The arrangement of individual vocabulary items on this map visualises the semantic and syntactic relationships encoded in the high dimensional embedding data [21].

To visualise the relationships between vocabulary items, we project a subset of forty-five whole-phrase bigrams belonging to the primary symptoms of COPD, namely dyspnea (breathlessness), cough and sputum. We then add the projections corresponding to a further 15 bigram phrases describing affective states such as *feel depressed* and *be embarrassing*.

Figure S1 shows the resulting two-dimensional map capturing the spatial relationship between self-reported symptoms and terms describing affective states. Whole-phrase bigrams express meaning less ambiguously than do single word unigrams. As an example of this, compare the words embarrassing and breathless with whole-phrase bigrams be-embarrassing and become-breathless. Bigrams therefore contribute to the map being more expressive.

Bigrams corresponding to the symptoms breathlessness, cough and mucus hypersecretion are arranged in distinct clusters such that there is no overlap between the clusters hosting the three symptom types. This is noteworthy, because the neural network is not explicitly trained to distinguish between dyspnea, cough and mucus hypersecretion.

The mucus and breathlessness clusters are located further from the cluster of affective states than the cough cluster. Mucus and breathlessness phrases share similar values on the second principal component. The two clusters are distributed broadly across the first principal component.

Vocabulary items corresponding to affective states, such as *feel depressed* and *experience anxiety*, are also organised into a well-defined cluster. The cluster occupies a region in the upper third segment of the map. Relative to the symptom clusters, the cluster of affective states is closest to the cluster of cough symptoms.

To support the quantitative findings described thus far, we present selected text from the original corpus featuring combinations of symptoms and impact keywords (Table S4). These examples have been rephrased to prevent any chance of de-anonymization of the individuals included in our analysis. It is apparent from these examples that patients share, candidly, their beliefs and preferences.

**Table S4.** Examples of short excerpts of patient contributed content. The excerpts are selected manually from the corpus by first searching for documents containing specific keyword combinations. The excerpts provide a rich sense of the lived experience of disease.

| Symptom/impact | Selected snippets of content contributed by patients |
| --- | --- |
| COPD symptoms | Feeling tired, breathless, and coughing is bad enough. But I also have a wide range of other symptoms. |
| Fatigue impact | Even leaving my bed in the morning can make me feel exhausted and out of breath. |
| Exercise impact | Being short of breath is scary. I can understand your fear that you might overextend yourself when out doing some exercise. |
| Mucus, cough, and impacts associated with social stigma | Coughing all the time impacts my social life and stops me from sleeping properly. |
|  | It has been an awful night, painful and embarrassing. |
|  | Coughing up mucus all the time can be embarrassing and disrupt a normal social life |
